# Post-mortem diagnosis of malaria in decomposed and embalmed body

**DOI:** 10.1007/s12024-024-00925-1

**Published:** 2024-12-09

**Authors:** Magdalena Kusior, Danuta Piniewska-Róg, Marta Wojtas, Marek Sanak, Martyna Maciów-Głąb, Artur Moskała

**Affiliations:** 1 Chair and Department of Forensic Medicine, Jagiellonian University Medical College, Kraków, Poland; 2 Department of Internal Medicine, Faculty of Medicine, Jagiellonian University Medical College, Kraków, 30-688 Poland

**Keywords:** Malaria, Autopsy, Post-mortem microbiological molecular diagnostics, Embalming

## Abstract

The diagnosis of malaria during the autopsy of a decomposed corpse may prove challenging. Macroscopic changes are non-specific and may include, among others, cerebral oedema, pulmonary oedema, hepatosplenomegaly and, on occasion, the presence of petechiae. The most effective diagnostic tools for malaria are the examination of blood smears and the use of rapid immunochromatographic tests. As a result of the progressive putrefaction of the corpse and blood hemolysis, classical tests are no longer viable. Consequently, the sole remaining option is the utilisation of real-time reaction (RT-PCR) to ascertain the presence of plasmodium DNA in specific organs. This study concerns the diagnosis of a fatal form of cerebral malaria in a 23-year-old Caucasian male who had travelled to Africa. The autopsy was conducted at a local hospital, after which the body was embalmed and stored in cold storage for a period of 8.5 months. Subsequently, the corpse was transported to Poland, where a further forensic autopsy was conducted. A significant challenge was to confirm the presence of malaria in a corpse that had been embalmed several months prior to the investigation. Samples were obtained from internal organs for genetic analysis to determine the presence of parasite DNA. An RT-PCR test was conducted on genetic material obtained from the brain, heart, lungs, kidney, liver, and spleen. The presence of Plasmodium falciparum genetic material was identified in samples obtained from the brain, lungs, kidney, liver, and spleen. These findings substantiated the post-mortem diagnosis of a severe form of cerebral malaria, which was the underlying cause of death.

## Introduction

Malaria is one of the most serious infectious diseases, along with HIV infection and tuberculosis, caused by one or more of five species of single-celled protozoa belonging to the genus Plasmodium. The most dangerous for humans is Plasmodium falciparum, which causes tropical malaria [1]. This protozoan is responsible for the majority of deaths resulting from this disease, both among the population residing in malaria-infected areas and among travellers [2]. The infection is transmitted by mosquitoes and is endemic, occurring primarily in certain regions of Africa, North and South America, and Asia [3]. Globally, it is estimated that 300–500 million new cases emerge annually, resulting in 1.5 to 2.7 million deaths [4].

It is estimated that approximately 10,000 cases of malaria are reported in Europe each year, primarily among recent travellers. In Poland, the number of cases does not exceed 50 per year [4].

The disease course can be variable, and the clinical presentation of the disease can range from asymptomatic to mild and uncomplicated, to severe and complicated, or even result in death. In general, the disease is classified into two distinct categories: uncomplicated or severe [5, 6]. Dormont et al. demonstrated a significant correlation between the clinical severity of symptoms among patients infected with Plasmodium falciparum and the parasite load, as determined by both microscopy and PCR [7]. It may therefore be the case that quantitative PCR is a useful tool for predicting the outcome.

It is acknowledged that there have been attempts to utilise the quantitative PCR technique in the diagnosis of infections in a forensic setting. This type of diagnostic tool can be employed to diagnose sepsis and assess the aetiology of inflammatory changes, particularly in neuroinfections, pneumonia, heart inflammation, food infections, and fungal superinfections [8, 9]. The aforementioned factors may prove crucial in verifying intrahospital infections, as well as in medical malpractice lawsuits [10]. 

The signs and symptoms of uncomplicated malaria cases are non-specific and may include fever, shivering, arthralgias, myalgias, headache, abdominal pain, nausea, vomiting, diarrhoea and fatigue [5]. Consequently, the clinical presentation may be atypical and susceptible to misdiagnosis as a “flu-like” illness [11].

A multitude of the clinical manifestations of severe malaria are attributable to the parasitized red blood cells (RBCs) adhering to the endothelial cells that line the small blood vessels, a process known as “cytoadherence.” This phenomenon results in the formation of small infarcts, capillary leakage, and organ dysfunction. In such instances, the presenting symptoms may include altered consciousness, seizures, severe anaemia (e.g. massive intravascular haemolysis), hypoglycaemia, respiratory failure or acute respiratory distress syndrome (ARDS), coagulopathy which may manifest with disseminated intravascular coagulation, metabolic acidosis, circulatory collapse, renal failure, haemoglobinuria, hepatic failure. Physical findings may include pallor, petechiae, jaundice, hepatomegaly, and/or splenomegaly [5].

This study presents a case of fatal cerebral malaria that was confirmed 8.5 months after death through a post-mortem examination and molecular malaria testing of an embalmed male.

## Case presentation

The case study concerns a 23-year-old Caucasian male of Polish nationality who was employed in one of the countries on the east coast of Africa. A review of the medical documentation and case files revealed that prior to travelling abroad, the patient had not been diagnosed with any significant diseases or chronic conditions and was not taking any medications on a regular basis. Prior to his departure, the patient was provided with medical advice at the Clinic of Tropical Diseases, which included guidance on malaria prophylaxis and the prevention of traveller’s diarrhoea. It was recommended that the patient use repellents (Mugga 50%) and Malarone (proguanil hydrochloride/atovaquone), among other measures. The patient was instructed to commence treatment with Malarone one day prior to departure and to continue taking it for a period of seven days following the conclusion of their stay.

In the eighth month of his stay, he presented with symptoms of illness, fatigue, and respiratory infection. The patient presented with a fever and abdominal discomfort. Over the following days, his condition deteriorated significantly, necessitating hospital admission. Upon admission, the patient was unconscious and febrile. Imaging diagnostics (chest X-ray and head CT) were performed but did not show any pathology. Laboratory tests confirmed the presence of severe malaria in the patient, caused by the parasite Plasmodium falciparum. Parasitemia was found to be 1.8% of red blood cells. Furthermore, laboratory tests yielded abnormal results, indicating the presence of severe intravascular hemolysis (anaemia, hyperbilirubinemia, elevated LDH), coagulopathy (thrombocytopenia, prolonged APTT), electrolyte imbalance (hypercalcemia, hyperkalemia, hypermagnesaemia, hyperphosphatemia, hyponatremia), and evidence of severe renal and hepatic failure, hyperuricemia. A summary of the selected test results is presented in Table 1.

The antimalarial treatment was initiated with artezunat. The patient remained unconscious (Glasgow Coma Scale score of 7/15) and subsequently developed severe respiratory failure shortly after admission to the hospital. Additionally, an episode of upper gastrointestinal bleeding and convulsions were observed during the initial hours of hospitalization. The treatment regimen was augmented with the addition of anticonvulsants, gastroprotective agents, antibiotics, and high-flow oxygen therapy. Despite the administration of treatment, the patient’s condition continued to deteriorate. On the second day of hospitalization, the patient experienced a sudden cardiac arrest, and resuscitation was unsuccessful.

A forensic medical autopsy was conducted on the day of the patient’s death. No injuries were identified, apart from the typical consequences of medical rescue operations. The autopsy report, which was included in the case files, was cursory in nature, yet it did describe several abnormalities. The presence of pleural and peritoneal effusions was documented, along with the diagnosis of bronchopneumonia, cardiomegaly, hepatomegaly, and acute fatty pancreatitis. The spleen was enlarged and exhibited the hallmark features of tropical splenomegaly syndrome (TSS). The analysis of the blood sample revealed a significant presence of malaria parasites.

The cause of death was determined to be severe malaria caused by a Plasmodium falciparum infection, accompanied by uremic encephalopathy, TSS, cardiomegaly, hepatomegaly, acute fatty pancreatitis, severe anaemia and acute renal failure.

Following the autopsy, the corpse was embalmed and stored in cold storage for 8.5 months in the African country due to logistical challenges associated with transporting the body to Poland. The specific embalming conditions remain unknown.

Following the transfer of the corpse to Poland, the prosecutor decided that a further autopsy was required to confirm results of the first postmortem examination.

The body exhibited indications of advanced putrefaction and exhibited the hallmarks of a prior autopsy, in addition to vascular access points utilized for the embalming of the corpse through infusion.

The cranial cavity had not been previously dissected, thereby allowing for an assessment of the brain structures. However, due to the advanced state of decomposition, a thorough examination was not possible. It was only possible to make a general statement that no focal discolourations were present. The macroscopic evaluation of individual organs was significantly limited by the effects of decomposition, embalming and the number of previous dissections. The only confirmed finding was splenomegaly. The atria and ventricles of the heart and the large blood vessels were empty, and it was not possible to obtain blood samples.

The main challenge for the autopsy was to re-confirm parasite infection. Due to the advanced stage of decomposition, immunochromatographic tests were ruled out, and the lack of blood made it impossible to perform standard parasitological tests. A large number of samples were taken from internal organs for toxicological and genetic examinations.

Liver and kidney samples were subjected to toxicological analysis for the presence of ethanol, methanol, isopropanol and acetone using gas chromatography (GC) with flame ionisation detection (FID). The toxicological examination revealed the presence of ethyl alcohol in concentration 0.3‰ (liver) and 0.2‰ (kidney). This finding could be fully explained by the advanced post-mortem processes and did not provide sufficient evidence to assume that the deceased was under the influence of ethyl alcohol at the time of death. Furthermore, the post-mortem material that was secured contained methyl alcohol (0.7‰ in the liver and 1.1‰ in the kidney), which is frequently employed for the embalming of corpses. However, the documentation did not include any information regarding the composition of the preservative agents used. A review of the medical records from the hospital treatment did not provide any evidence to suggest that the patient had suffered from methanol poisoning.

Toxicological analysis of internal organ samples was conducted using the LC–ESI-MS–MS method according to standard protocol using in our Toxicological Departament and previously described by Rojek et al. [12] The internal organ samples (liver and kidney) revealed the presence of substances with anticonvulsant (diazepam), analgesic (acetaminophen, dipyrone), antimalarial (quinine) and antiarrhythmic (lidocaine) properties, which corresponded to the records in the submitted medical documentation.

Toxicological analysis of hair samples of the deceased’s hair was conducted using the LC–ESI-MS–MS method previously described by Klys et al. [13]. The drugs were identified in the analysed segment of hair, along with the additional presence of tramadol. No antimalarial drugs were identified. The main objective of the multiparameter analysis was to identify the presence of antimalarial drugs, including atovaquone, proguanil, quinine, and a comprehensive range of antibiotics (e.g. doxycycline). On the other hand, it should be emphasisedthat toxicological analysis of hair samples is aimed to prove long-time intake, rather than to identify a one-time intake. The absence of any antimalarial medications in the analysed hair samples ruled out that the patient did used the pharmacological prophylaxis of malaria in the final months of his life. As indicated in the literature, antimalarial drugs are incorporated into the hair structure and can be successfully detected using the LC-MS-MS method [14]. 

**Fig. 1:**
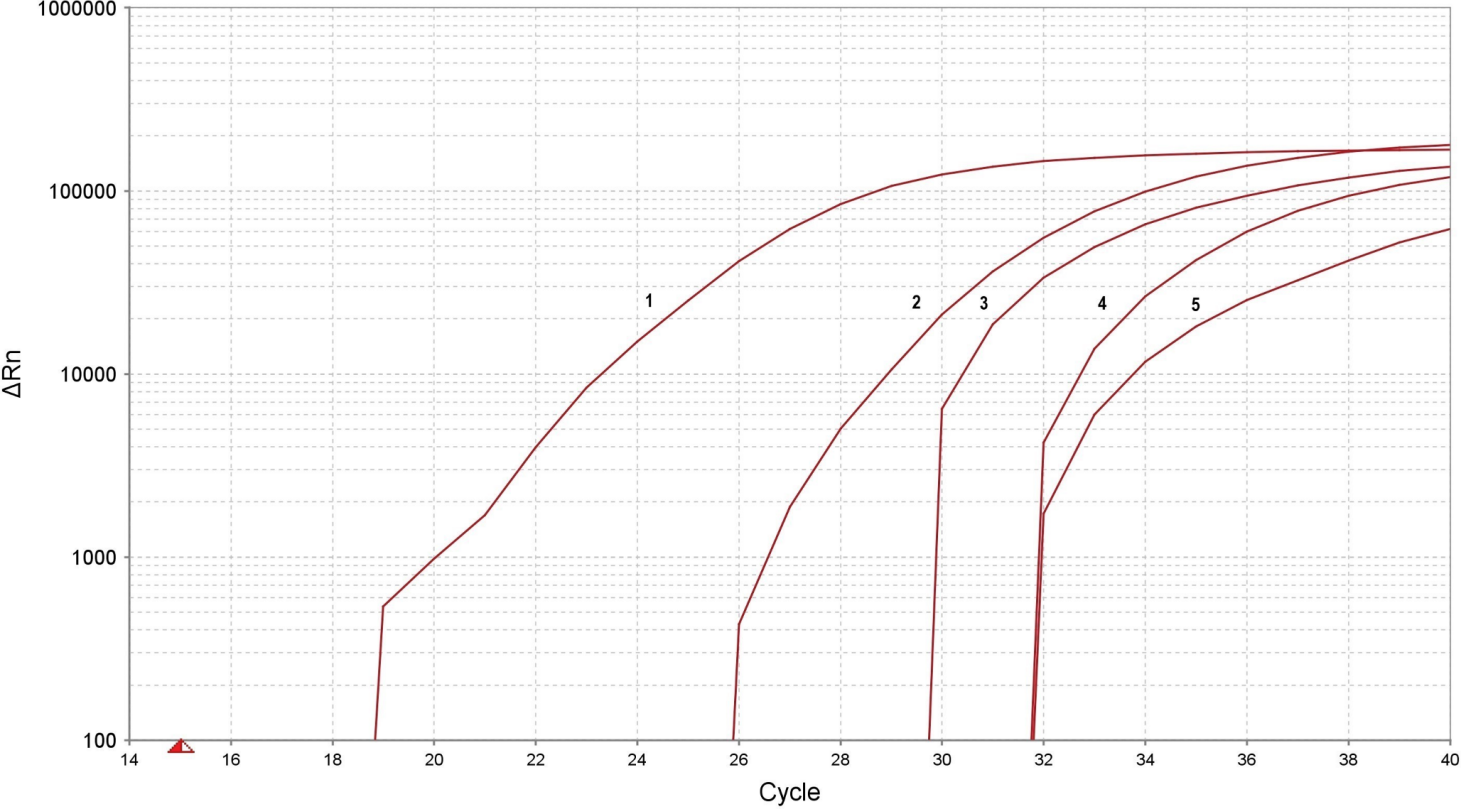
Real-time PCR amplification plot for malaria detection of postmortem samples: positive control (**1**), kidney (**2**), brain (**3**), lung (**4**) and liver (**5**), showing the log of the increase in the fluorescence plotted versus cycle number (ΔRn vs. Cycle). Malaria DNA detection in the samples were detected at average of duplicate material at the threshold cycle (Ct): positive control (Ct = 18.170), kidney (Ct = 25.809), brain (Ct = 29.278), lung (Ct = 31.299) and liver (Ct = 31.439)

In the present case, the most pertinent information was derived from molecular tests. Post-mortem samples from a range of organs, including the heart, lung, brain, kidney, liver and spleen, were subjected to examination. The total genomic DNA was extracted using a silica-based method with a Sherlock AX kit (A&A Biotechnology, Poland), and the DNA amplification was performed on a QuantStudio 5 Real-Time PCR System (Applied Biosystems, USA) with the real-time PCR (RT-PCR) method using a Malaria TaqMan PCR (Norgen Biotek Corp., Canada) kit, in accordance with the manufacturer’s protocols. The Malaria TaqMan PCR Kit is designed for the detection of malaria-specific DNA using a real-time PCR based on the use of a 5’-nuclease assay of a TaqMan reporter probe. The molecular testing for malaria parasite infection revealed the presence of malaria parasite DNA in the lung, brain, kidney, liver and spleen sections. Conversely, no malaria parasite DNA was detected in a sample obtained from the heart. The results of the duplicate reactions for each tissue section were found to be consistently accurate. The correct result for both the positive and negative standards was obtained, as well as the internal control, in each diagnostic reaction. This was done to ensure the quality of the isolated DNA sample. The result of the molecular test was unequivocal, confirming a severe infection with the malaria parasite in its most severe form, cerebral malaria. Figure 1.

## Discussion and conclusion

Malaria is the most prevalent fatal parasitic disease worldwide. The diagnosis of malaria in post-mortem examinations is typically conducted through a combination of classical methods, including macroscopic examination, microscopic examination of sections from specific structures, and parasitological examination of blood samples. Additionally, rapid immunochromatographic tests are employed. PCR-based methods, such as real-time PCR, multiplex PCR, and nested PCR, offer a highly accurate and sensitive approach for detecting low-level malaria infections in patients. These methods have been shown to have excellent specificity and sensitivity, making them an invaluable tool in the diagnosis of malaria [15–16].

Malaria can be suspected based on the patient’s travel history, clinical symptoms and the physical findings at examination. Nevertheless, a definitive diagnosis necessitates the demonstration of a positive result through the utilisation of laboratory tests, such as light microscopy of blood smears and rapid diagnostic tests for malaria parasites or their components. The World Health Organization (WHO) recommends these methods as the gold standard for detecting symptomatic malaria infections. However, they have limitations in asymptomatic patients, especially when parasite loads are low. Polymerase chain reaction (PCR) methods, including quantitative reverse transcription-PCR (RT-PCR), have become the most frequently employed molecular techniques for malaria diagnosis and Plasmodium species identification due to their sensitivity and specificity [5, 16–21].

Boonma et al. (2007) conducted a comparative analysis of three PCR-based techniques for the diagnosis and identification of Plasmodium falciparum, Plasmodium vivax and mixed infections, as measured by microscopy [22]. It was determined that real-time PCR was the sole technique that demonstrated 100% sensitivity and specificity for the diagnosis of each species and mixed infections. Molecular techniques demonstrated superior performance compared to traditional microscopic methods, which exhibited a false-negative rate of 9.6% [22]. At present, malaria PCR-based methods are a commonly employed molecular tool for confirming the species of the malaria parasite in living individuals following diagnosis by smear microscopy or RDT [19]. In the context of post-mortem diagnosis, it is frequently the sole method for identifying malaria infection [11, 23–25].

The post-mortem diagnosis of malaria may prove challenging, particularly when autolysis and microbiological contamination are advanced, especially in cases with an extended interval between death and post-mortem examination. In such instances, real-time PCR is regarded as the optimal approach for diagnosing malaria. In all cases of fatal malaria described by Berens-Riha et al. (2009), the post-mortem findings were unremarkable and did not suggest the final diagnosis [24]. The macroscopic findings were unspecific, while some of the histological and microscopic blood smear results were not evaluable. PCR and an immunochromatographic (ICT) test performed with cadaveric blood were the only methods that yielded positive results in all malaria patients.

In the case, the only means of confirming the diagnosis of severe cerebral malaria in post-mortem examinations was through molecular tests designed to search for the genetic material of Plasmodium falciparum in sections of individual organs, including the brain. The advent of highly sensitive molecular tests, such as real-time qPCR, has provided a means of detecting pathogen DNA sequences at a point in time when the performance of classic parasitological, microscopic, or immunochemical tests was no longer feasible. The utilisation of molecular tests employing the real-time qPCR technique has become increasingly prevalent in recent years, playing a pivotal role in the diagnosis of infectious diseases [26–28]. Due to the nature of the infection with the Plasmodium falciparum, which results in, among others, adhesion of RBCs to epithelial cells, the genetic material of the parasite can be detected in various internal organs. The organs with the highest diagnostic potential are those, with a well developed network of capillaries, e.g. the brain, lungs, kidneys, liver.

The formation of small clots in the mentioned internal organs promotes the accumulation of the genetic material of the malaria parasite, which makes it possible to detect the infection in the decomposed human body.

In the analyzed material, there were some differences observed in the parasite’s DNA content in individual organs. It’s probably resulted from the intravital distribution of the parasite’s genetic material. The quantity of malaria DNA (as displayed in Fig. 1) was not evaluated in conjunction with the state of decomposition of each organ, as the body had been previously autopsied and embalmed, what changed the pattern of the standard decomposition processes [29]. 

It should be noted that the deceased’s body had been autopsied 8.5 months earlier and then embalmed. During the (second) autopsy conducted in our Department, the corpse presented indications consistent with advanced putrefaction. The internal organs of the chest and abdominal cavity were dissected and placed loosely in the torso. In this case, the process of putrefaction was altered by previous autopsy and embalming. Also, the arrangement of internal organs was changed, which significantly affected post-mortem bacterial translocation. Due to the very large number of variable factors, in this case the quantity of malaria parasite DNA in individual internal organs was not compared to the state of their decomposition.

The presented case is the first recorded study to detect malaria in post-mortem samples even months after death from a heavily decomposed corpse and after embalming. Therefore, the passage of time, the severity of late post-mortem lesions, and embalming of the corpse do not preclude the possibility of post-mortem diagnosis of Plasmodium falciparum infection.

**Table 1:** Laboratory test result

Laboratory test result	
hemoglobin	33.6 g/dl
total bilirubin	10.64 mg/dl
direct bilirubin	7.54 mg/dl
LDH	1199U/l
PLT	37 000/microliter
APTT	54.6 s
Ca	9.4 mg/dl
K	5.48mmol/l
Mg	2.63 mg/dl
*P*	5.48 mg/dl
Na	133.4mmol/l
creatinine	2.96 mg/dl
eGFR	28 ml/min/1,73m^2^
urea	92 mg/dl
BUN	42.99 mg/dl
uric acid	7.99 mg/dl
AST	106.9U/l
ALT	88.3U/l
alkaline phosphatase	242U/l
CRP	> 120 mg/l

## Keypoints

1. Post-mortem microbiological molecular diagnostics of malaria.
2. The influence of post-mortem processes on the diagnosis of malaria in the post-mortem examination,
